# Motivational factors and financial incentives to mitigate tail biting in pig production in Finland

**DOI:** 10.3389/fvets.2026.1860638

**Published:** 2026-08-19

**Authors:** Minna Väre, Hilkka Koskikallio, Mari Heinonen, Miina Tuominen-Brinkas, Camilla Munsterhjelm, Anna Valros, Jarkko K. Niemi

**Affiliations:** 1 Natural Resources Institute Finland (Luke), Bioeconomy and Environment, Business Economics, Helsinki, Finland; 2 Department of Production Animal Medicine, Faculty of Veterinary Medicine, University of Helsinki, Helsinki, Finland; 3 Natural Resources Institute Finland (Luke), Bioeconomy and Environment, Business Economics, Seinäjoki, Finland

**Keywords:** economy, finishing pig, incentive, motivation, tail biting, weaner pig

## Abstract

Tail biting affects the health and welfare of pigs, the quality of pig carcasses, and the profitability of production in both weaner and finishing pig production. There are specific quality criteria for weaned pigs, and tail damage can lead to a reductions in the purchase price of weaner pigs as well as to carcass rejections at the slaughterhouse. Although this issue is important, research has paid little attention to tail biting in weaner pig production. The present study aimed to investigate the impact of various motivational factors and economic incentives on the management of tail biting on Finnish pig farms and to assess the costs of tail biting in weaner pig production. The data were collected by interviewing representatives of 15 farms producing finishing pigs and 10 farms producing weaner pigs during 2021–2022. The interviews covered risk factors for tail biting and the factors that most motivated producers to reduce the occurrence of tail biting. Additionally, using a calculation model, the economic significance of tail biting in nurseries was assessed. The interviewed farmers were very or fairly motivated to address tail biting when they observed it on their own farms. Half of the representatives from farms producing weaner pigs found that the management measures were laborious to implement. The economic incentives that most motivated farmers producing weaner pigs to address tail biting were obtaining a better price for intact-tailed (undocked tails with no tail lesions) pigs and financial support for measures to manage tail biting. Representatives of finishing farms were most motivated by the decrease in carcass condemnations and increase in meat prices. The results suggest that the desire to produce healthy pig with high welfare also motivated Finnish farmers. According to the calculation model and a hypothetical scenario, tail biting in weaner pig production can cause a loss of up to approximately €32 per bitten pig in Finland, taking into account possible effects during the finishing phase, reduced growth, treatment of the tail-damaged pig, and various income losses and additional costs. As the consequences of tail biting can be substantial, it is important that farmers are informed about the potential financial losses and impairment of animal welfare in the event of tail biting occurrence as well as possible means to mitigate and prevent tail biting.

## 1 Introduction

Tail biting is an important multifactorial animal welfare and economic problem in pig production (1). Based on a review, the costs of tail biting lesions across Europe are approximately €0.6 to €3.4 per finished pig (2). Tail lesions cause health issues in pigs, affect the quality of both pigs and pig carcasses, and decrease the profitability of pig production (1, 3, 4). Tail biting has been connected to increased stress levels in pigs (5), and multifactorial housing- and management-related risk factors for tail biting have been reported (3, 4).

Various risk factors can lead to biting incidents; therefore, multiple measures may be used to reduce the problem. Managing tail biting through measures such as increasing space allowance or providing more enrichment results in additional work and costs [e.g., (1, 3)]. Recent studies have, however, found a strong association between improved welfare conditions and enhanced farm productivity (1, 6, 7). Moreover, improvements in animal welfare could also be supported either by markets or by public policy, which provides producers with additional incentives (8).

One of the policy incentives aimed at enhancing pig welfare is the animal welfare compensation available to Finnish pig farms for improving the living conditions of weaner and finishing pigs [Common Agricultural Policy (CAP) plan for Finland 2023–2027]. By improving housing conditions and providing enrichment, it is possible to mitigate tail biting [see, e.g., (9)]. This may reduce antimicrobial usage as there are fewer tail lesion cases to treat. The Finnish animal welfare compensation provides farmers a payment that may cover the cost of additional work, enrichment, and other inputs used to improve pig welfare. The condition of the pigs and the quality of their care are assessed by the proportion of intact pigs. Meat inspection at the slaughterhouse records information about tail lesions, and the data are analyzed by an independent party (10). As one of the quality criteria for weaned pigs, tail damage can lead to a deduction in the price of the pig.

The routine tail docking of pigs has been prohibited in the EU since 2003 (11, 12). While some Member States, such as Finland, have prohibited tail docking completely, others have continued to allow the practice under exceptions permitted by EU regulation. The widespread use of tail docking seems to be accepted because the alternative measures that producers are required to take before resorting to it are not specified in detail and because it appears to be less risky and more profitable to produce docked than undocked pigs (2). Furthermore, Member States have increasingly developed plans to reduce or abandon tail docking for animal welfare and ethical reasons (1, 3). According to Valros et al. (4), most Finnish pig farmers did not consider tail biting as particularly severe problem, even though tail docking was not allowed. In Finland, tail docking has never been widely used. Similarly, farmers’ attitudes toward tail biting and tail docking were found to differ between farmers with different experiences of tail docking (4).

To decrease the risk of tail biting and improve pig welfare, more information is needed not only on the risk factors for tail biting but also on the incentives that motivate pig producers to manage tail biting on their farms.

While many studies have examined tail biting in finishing pig production, less attention has been paid to tail biting in weaner pig production. Hence, the aim of this study was to investigate the importance of various motivational factors, including economic incentives, for the management of tail biting on Finnish pig farms and to assess the financial costs of tail biting occurring in weaner pig production.

## 2 Materials and methods

### 2.1 Farm interviews

The data were collected by interviewing representatives from 15 Finnish farms producing finishing pigs and 10 farms producing weaner pigs during 2021–2022. None of the pigs were tail-docked. The farms included customers of three major slaughterhouse companies in Finland and were selected by convenience sampling. All farms were typical intensive Finnish pig farms. Pigs on all farms were reared in partly slatted pens with, on average, 20 weaner pigs or 11 finishing pigs per pen, with a space allowance of 1 m^2^ per finishing pig. On all farms, rooting material, such as straw or hay, turf, sawdust or paper, was used.

One of the authors interviewed either a farmer or a farm worker at each farm. The interviews consisted of 52 questions on the practices of the farm and the interviewee’s perceptions of tail biting. Eight of these questions were analyzed in this study, and these questions covered risk factors for tail biting, motivation to address tail biting, the factors that most strongly motivated producers to reduce tail biting, and the economic significance of tail biting and preventive measures. In addition, interviewees were asked about their attitude toward tail docking. The interviews were recorded and transcribed. The data were analyzed qualitatively (thematic analysis) and by quantifying the occurrence of various aspects and summarizing participants’ views.

The interview included questions rated on a 7-point Likert scale. First, interview participants were asked about their motivation to manage tail biting on their own farm using a 7-point scale, with a score of 1 indicating that they were not motivated and 7 indicating that they were very motivated. Second, interview participants were asked how difficult or laborious they considered managing tail biting on their farm Using a 7-point scale, with a score of 1 indicating that managing tail biting was not difficult or laborious and 7 indicating that managing tail biting was very difficult or laborious. Third, interview participants were asked how severe they consider tail biting as an animal welfare and financial issue. A Likert scale from 1 to 7 was used, with 1 indicating not a severe problem and 7 indicating a very severe problem.

Finally, interviewees were asked to what extent different factors would motivate them to manage tail biting and to rank these factors accordingly. Motivational factors presented to all interviewees included pig welfare, pig health, a sense of success when there was no tail biting, an increase in the pork producer price, welfare compensation for managing tail biting, knowledge (research) or training on tail biting management, support for systematic tail biting management, good experiences from other farms with tail biting management, industry’s good image in the eyes of consumers, veterinarians’ recommendations for more accurate tail biting monitoring/prevention, the serious attitude of outsiders toward tail biting, and a record-keeping obligation for tail biting. Interviewees producing finishing pigs were also asked about reducing carcass rejections and intact tail support, and farmers producing weaner pigs were asked about price reductions for bitten pigs and better prices for intact-tailed pigs. Factors were graded using a Likert scale from 1 to 7 (1 = does not motivate; 7 = motivates very much). In the analysis, different types of motivational factors were clustered into four aspects in order to compare their importance in motivating farmers to manage tail biting. These four clusters were factors related to (1) animal health and welfare, (2) economics, (3) information and support for preventing tail biting, and (4) the influence of third parties or different obligations.

Interviewees’ responses to each categorical question were presented graphically, and the responses were compared qualitatively between questions and farm types. Because of the small number of observations, the statistical significance of responses between farm types and questions was not tested. Rather, the interview data were considered qualitative data that identified issues raised by farmers.

### 2.2 Calculation model

A model to calculate the economic significance of tail biting in weaner pig production was developed. The model included a gross margin calculation for a farrowing-to-finishing operation. Although it simulated production costs and revenues per produced slaughter pig (i.e., costs from birth to slaughter), the examined impacts of tail biting were associated with lesions originating during the weaner production stage. The model considered production costs and performance, the incidence of bitten tails, and the impact of tail biting risk on pigs’ growth, sale value, feed consumption, mortality, and carcass condemnations. The model was developed in Excel, and it utilized data from another study (8).

Financial losses and costs resulting from tail biting incidents include additional labor, feed, veterinary, and other costs resulting from impaired production performance, treatment, and care of bitten pigs and other pigs [see (2)]. These losses are influenced by the incidence and severity of tail biting, which depend on the context, such as the risk factors for tail biting are present on the farm. Revenues are also lost primarily because of reduced growth and increased mortality risk among bitten pigs. These effects are likely to lead to impair the feed conversion ratio, as slower growing pigs may use a higher proportion of their diet for body maintenance functions. Besides tail biting itself, the measures used to prevent or control in tail biting are costly.

A baseline scenario and sensitivity analysis scenarios for a pig farm in Finland were simulated. In the baseline scenario, bitten pigs were assumed to experience, on average, an 11% reduction in daily weight gain during weaner rearing [see (8, 13)]. The cost of treating bitten pigs was estimated at €12.03 per pig, and the cost of rendering a dead pig was estimated at €4.27 per weaner pig. These costs included medicines, veterinarians’ and farmers’ working time, pen enrichments, and the cost of rendering dead pigs and were based on expert information on treatment needs and on the prices of veterinary services, medicines, and rendering costs obtained from veterinary service providers, medicine suppliers, and the rendering company Honkajoki Ltd. Data on feed prices and output prices were based on information obtained from feed companies and slaughterhouses in Finland. Based on information from the farms, pig mortality was assumed to increase by 6.6 percentage points among bitten pigs. Pigs with tail damage were considered to face a 0.9 percentage point increase in carcass condemnations. The key parameters of the baseline analysis are presented in Supplementary Table S1. In the sensitivity analysis, scenarios in which feed prices, the price of pigmeat, the cost of treating bitten pigs, or mortality-related costs were increased by 20%.

The costs and revenues were calculated per pig for bitten and non-bitten pigs. The financial impact of tail biting was calculated as the difference between these two estimates. Based on data collected from a sample of five interviewed farms, three hypothetical *what if*-scenarios illustrating the significance of the prevalence of tail biting in weaner pigs were postulated and then simulated. In the *baseline scenario*, 5.6% of pigs were assumed to have a tail lesion requiring treatment. This already represents a relatively high prevalence and suggests that the threshold for initiating treatment on pigs was quite low. In a *high tail biting risk* scenario, 10% of pigs were assumed to have a tail lesion requiring treatment. In the third scenario, which described an *escalated tail biting outbreak*, up to 56% of pigs were assumed to have tail lesions requiring treatment.

## 3 Results

### 3.1 Risk factors

Interviewees identified several risk factors for tail biting. A majority of risk factors were related to feeding or housing conditions. Changes in feeding or the feeding system, as well as technical problems with feeding or air-conditioning systems, may cause stress in pigs. Changing weather conditions, particularly during spring and autumn, may cause changes in lighting, temperature, or other environmental conditions for pigs. Interviewees from farms producing weaner pigs suggested that there may be too little space for feeding, especially at the end of the rearing phase. Similarly, mixing of animals, especially combining litters, may increase stress and the risk of tail biting among weaner pigs. A lack of enrichment and poor quality pigs were considered risk factors for tail biting, especially on farms producing finishing pigs.

Tail biting was considered to increase the risk of diseases and removal of pigs. The need for medication and additional enrichment increased labor requirements and costs. Extended duration of rearing increases production costs and can lead to a shortage of pen space, further increasing the risk of tail biting. In addition, reduced quality of pigs led to price reductions and increased carcass rejections, and both of which reduced the profitability of production.

Maintaining good housing conditions and feeding practices, together with sufficient enrichment use and continuous monitoring of pigs, was considered as the most relevant approach to managing tail biting. Nearly all interviewed farmers producing weaner pigs and some interviewed farmers producing finishing pigs considered that producing pigs without tail docking provided an image benefit. A majority of the interviewees did not think that tail docking should be allowed in Finland, even though they knew that it is still permitted in some countries.

### 3.2 Motivation

**Figure 1:**
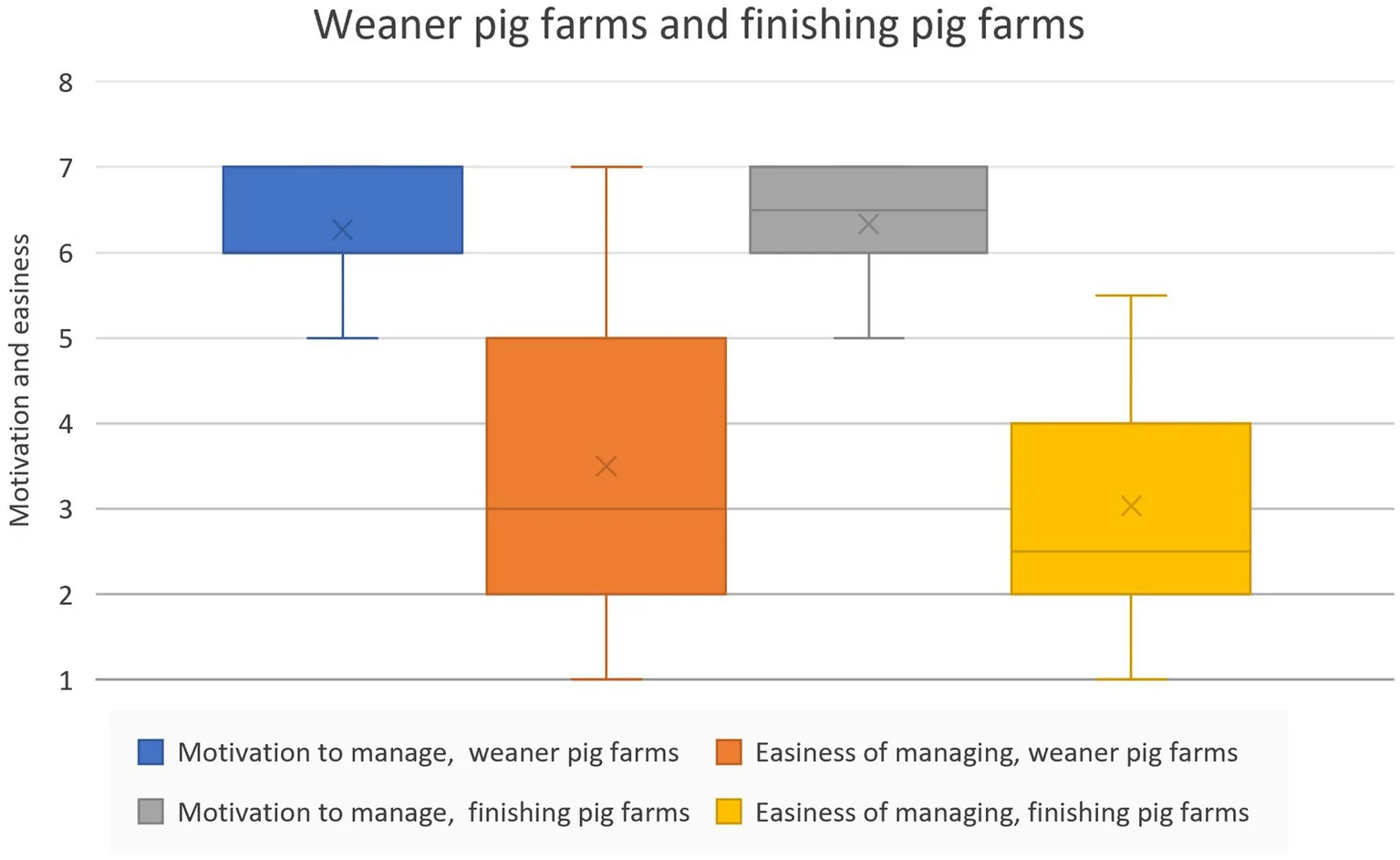
Weaner pig (*N* = 10) and finishing pig (*N* = 15) farmers’ motivation to manage tail biting on their farm (1 = not motivated, 7 = very motivated) and their views on easiness of managing tail biting (1 = not difficult to manage, 7 = difficult to manage).

All interviewees were very or fairly motivated to address tail biting when they observed it on their own farm (Figure 1). Half of the interviewees producing weaner pigs found the management measures laborious and difficult to implement (Figure 1), whereas farmers producing finishing pigs found the implementation of management measures somewhat easier and less laborious (Figure 1).

**Figure 2:**
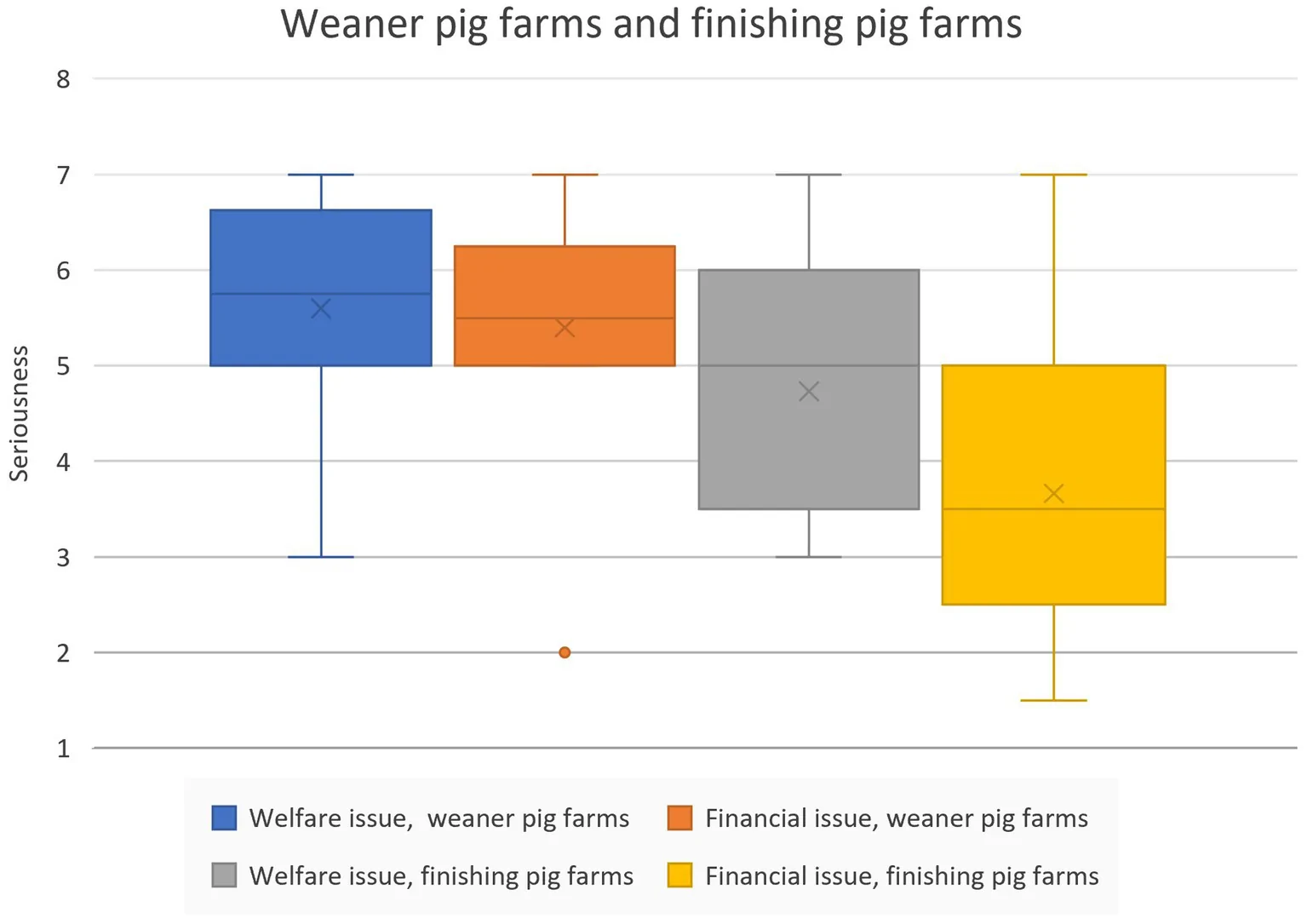
Weaner pig (*N* = 10) and finishing pig (*N* = 15) farmers’ views on how severe an animal welfare and financial issue tail biting is (1 = not a severe problem, 7 = very severe problem).

Farmers producing weaner pigs considered tail biting to be both a severe or very severe animal welfare issue and a severe or very severe financial issue (Figure 2). The views of farmers producing finishing pigs were somewhat more divided (Figure 2) than the views of farmers producing weaner pigs. Nearly half of the farmers producing finishing pigs considered tail biting to be a fairly severe or severe welfare issue but only a third of them considered it a fairly severe or severe financial issue.

### 3.3 Incentives

To compare the importance of different motivational factors for intervening in tail biting, the factors were clustered into four aspects. In the analysis, the following factors related to animal health and welfare were considered: pig welfare, pig health, and sense of success when there was no tail biting. Economic factors included increases in the producer price of pigmeat, welfare compensation for tail biting management measures, reduced carcass rejections, support for intact tails, price reduction for bitten pigs, and higher prices for intact-tailed pigs. Factors related to information and support for managing tail biting were knowledge from research, training on tail biting management, support for systematic tail biting management, and positive experiences from other farms in tail biting management. Factors related to the influence of third parties or different obligations were the veterinarian’s call for more accurate tail biting monitoring or prevention, the serious attitude of outsiders toward tail biting, record-keeping obligation for tail biting, and the pig industry’s good image among the eyes of consumers.

**Figure 3:**
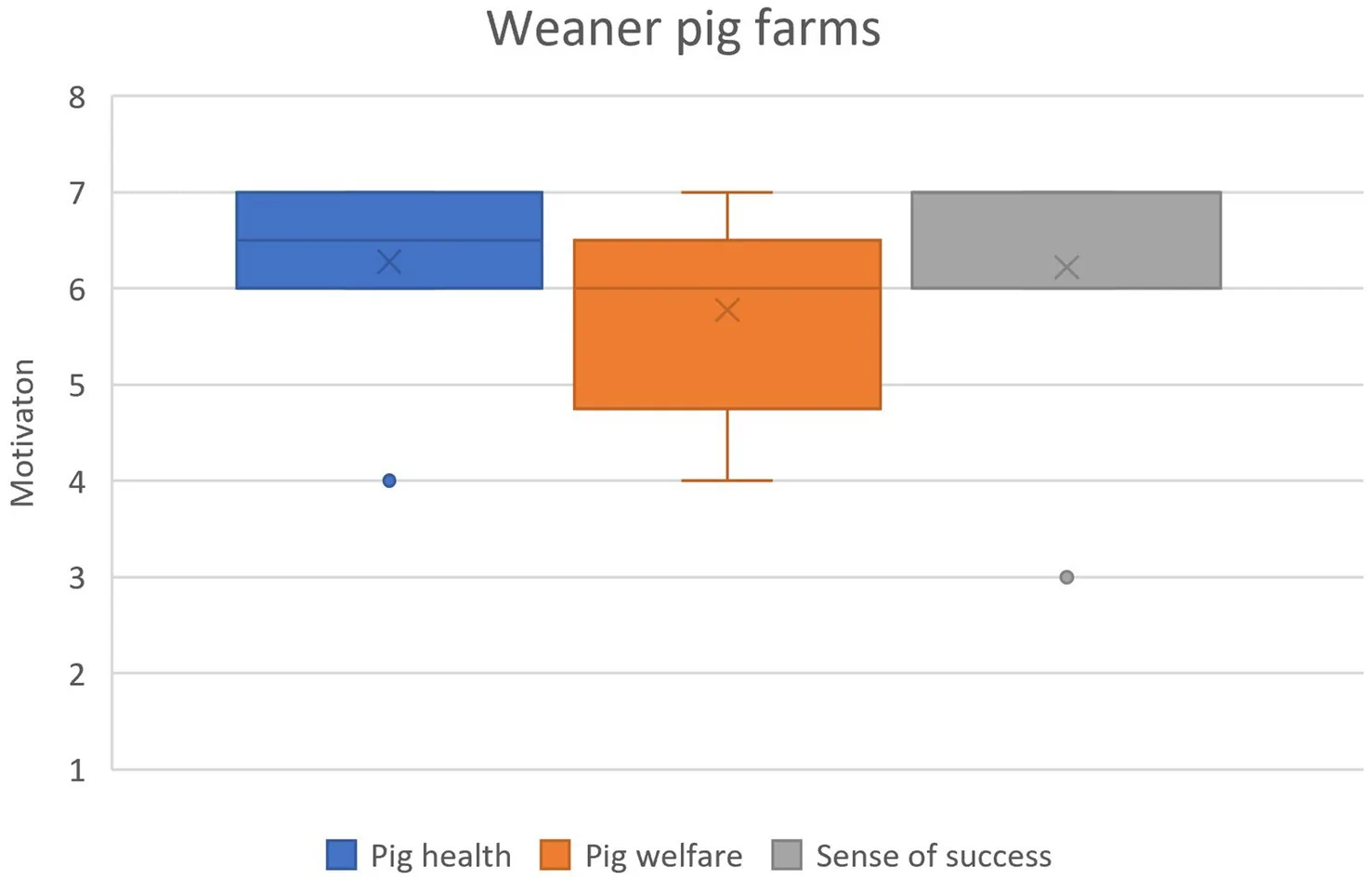
Weaner pig farmers’ (*N* = 10) views on how much different animal health and welfare related factors would motivate them to manage tail biting (1 = does not motivate, 7 = motivates very much).

**Figure 4:**
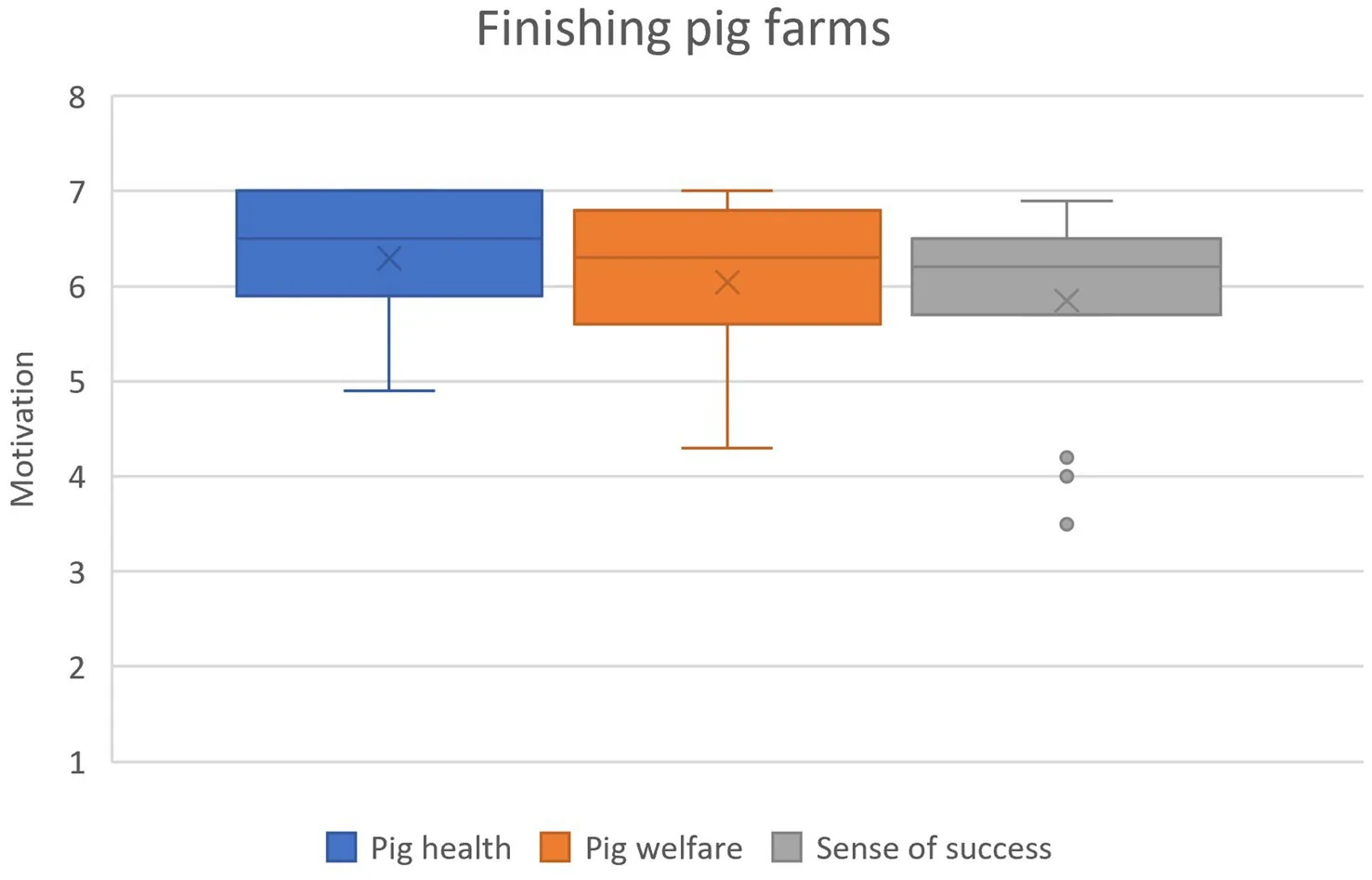
Finishing pig farmers’ (*N* = 15) views on how much different animal health and welfare related factors would motivate them to manage tail biting (1 = does not motivate, 7 = motivates very much).

**Figure 5:**
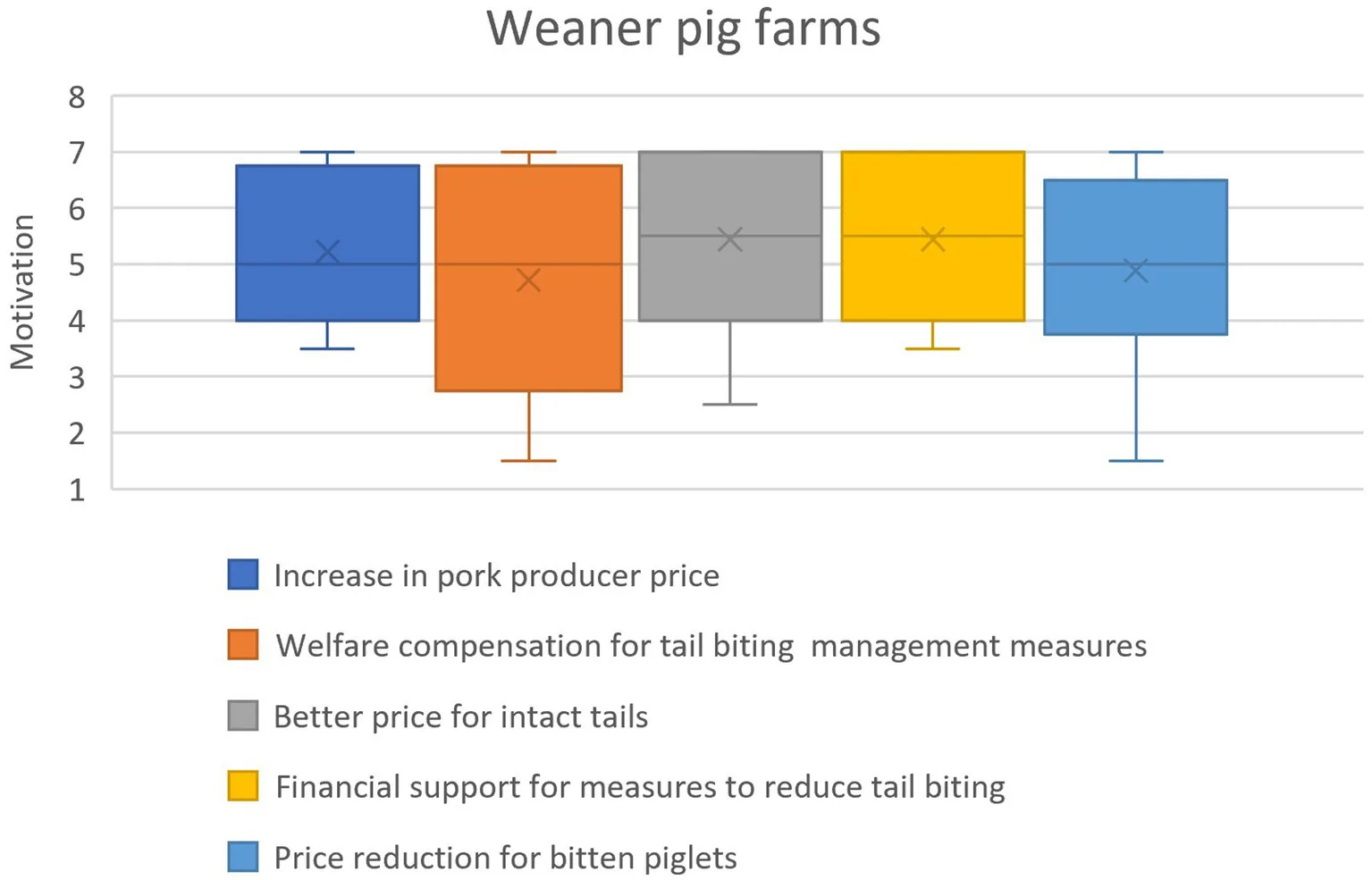
Weaner pig farmers’ (*N* = 10) views on how much different economic factors would motivate them to manage tail biting (1 = does not motivate, 7 = motivates very much).

**Figure 6:**
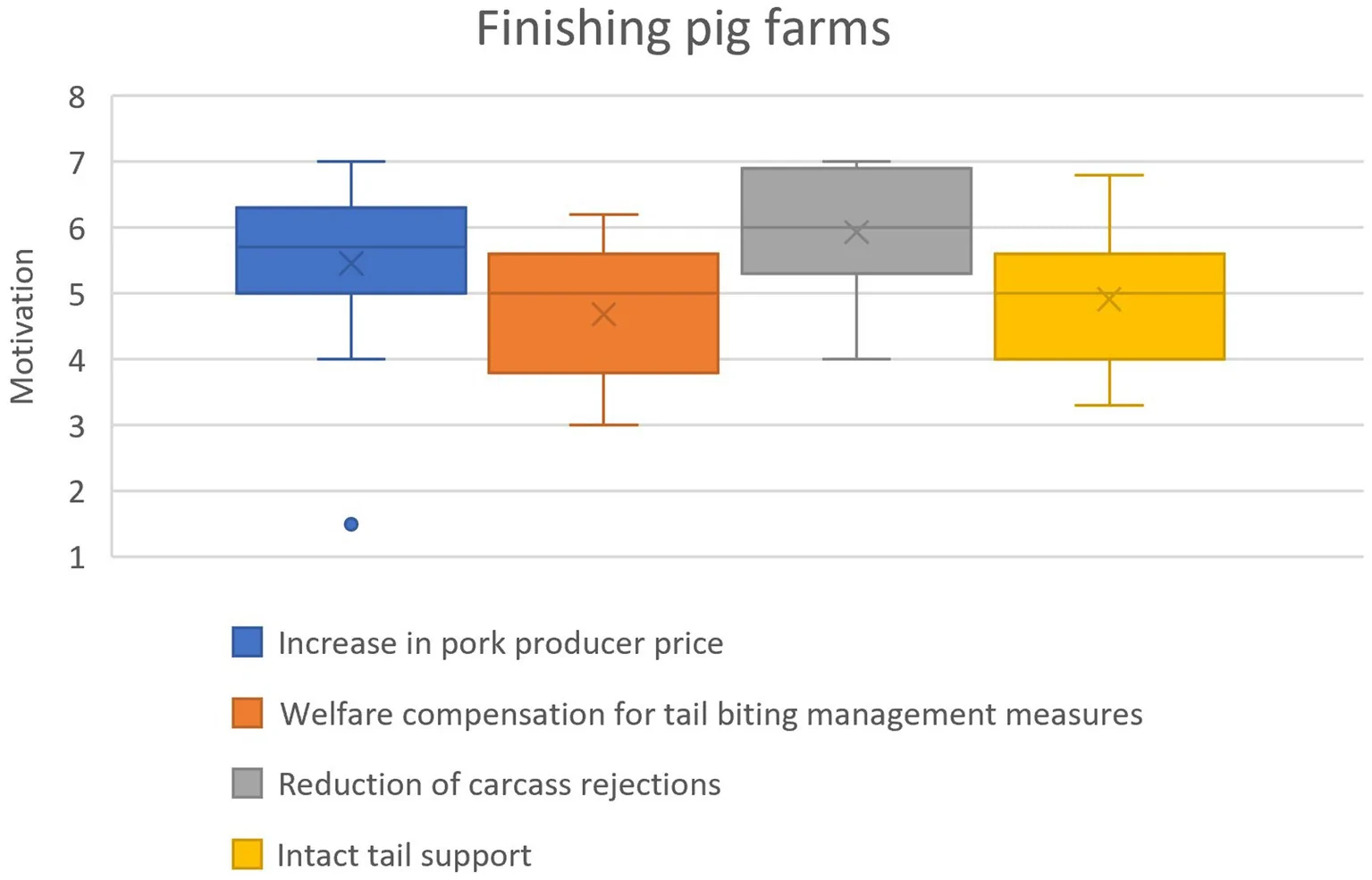
Finishing pig farmers’ (*N* = 15) views on how much different economic factors would motivate them to manage tail biting (1 = does not motivate, 7 = motivates very much).

According to the interview data, pig health and welfare and economy-related factors motivated Finnish farmers to manage tail biting outbreaks more than factors related to information and support received for managing tail biting, the influence of third parties, or the various obligations imposed on farms (Figures 3–6 and Appendices 1, 2).

Pig health and a sense of success when managing to rear high-quality, intact-tailed pigs motivated farmers producing both weaner pigs and finishing pigs very much (Figures 3, 4). Furthermore, pig welfare motivated farmers very much, although especially the views of farmers producing weaner pigs were diverse (Figures 3, 4).

The economic incentives that motivated farms producing weaner pigs to address tail biting the most were higher prices obtained for intact-tailed pigs and financial support for management measures. An increase in the price of pigmeat and financial support for measures to reduce tail biting also motivated farmers slightly more than price reductions for pigs with tail damage (Figure 5).

Finishing farms were most motivated by reductions in carcass condemnations and increases in the price of meat (Figure 6). Welfare compensation for measures to manage tail biting and intact tail support motivated farmers to a slightly lesser extent (Figure 5). Overall, the views of farmers producing weaner pigs were more divided than were the views of farmers producing finishing pigs (Figures 5, 6).

Knowledge and training on the management of tail biting motivated farmers producing finishing pigs only to a limited extent to mitigate tail biting, whereas farmers producing weaner pigs found them rather motivating (Appendix 1). Farmers producing weaner pigs found support for systematic tail biting management more motivating than did farmers producing finishing pigs (Appendix 2). Positive experiences of other farmers reported both farmers producing weaner and finishing pigs moderately (Appendices 1, 2).

The pig industry’s good image among consumers motivated interviewees to control tail biting much or very much (Appendix 2), whereas veterinarian’s recommendations for more accurate tail biting monitoring or management, the serious attitudes of external stakeholders towards tail biting and obligation to keep records on tail biting motivated farmers to a lesser extent. Furthermore, interviewees’ views on these motivational factors were quite heterogeneous (Appendix 2).

### 3.4 Economic significance

According to the computational model and simulated what-if scenarios, tail biting in weaner pig production can cause a financial loss of up to approximately €32 per bitten pig in Finland when possible long-term consequences in the finishing phase are taken into account (i.e., the delayed consequences of tail biting incident that occurred during weaner pig production, but not consequences of tail incident that occurred during the finishing stage). These losses arise from reduced growth, treatment of the tail-damaged pig, and various income losses and additional costs. The contribution of mortality and treatments of weaner pigs to the financial loss was €12.94 per bitten pig. Changes in production efficiency, such as reduced growth rates, caused a €14.05 financial loss per bitten pig.

The financial loss was increased by €0.80 when feed prices increased by 20%. A 20% increase in the price of pigmeat raised the financial loss by €0.5; a 20% increase in treatment-related costs by €0.53; and a 20% increase in the costs of mortality by €2.53 per bitten pig.

**Figure 7:**
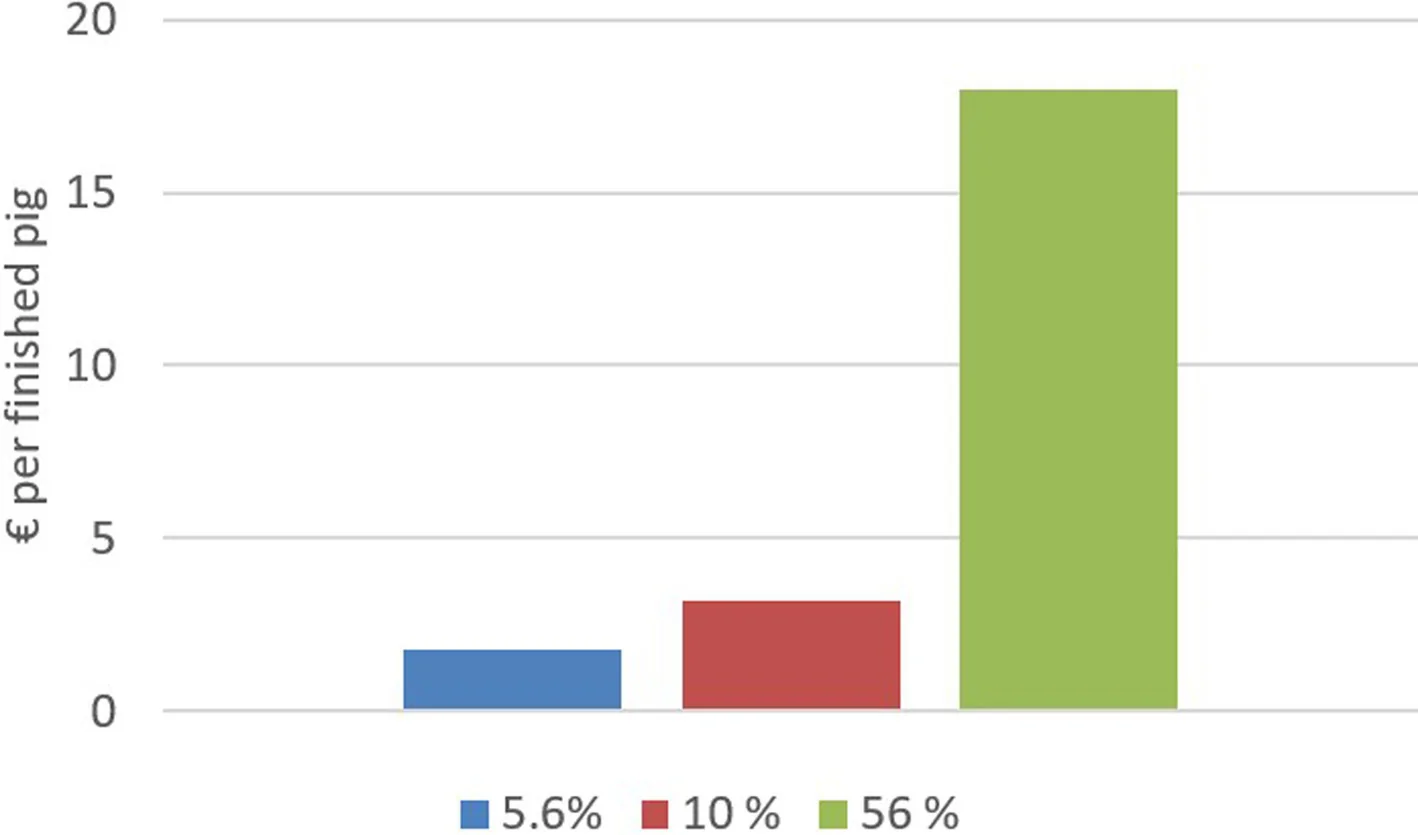
Financial loss (€/bitten weaned pig) when 5.6% (baseline scenario), 10% (high tail biting risk scenario), or 56% (escalated tail biting scenario) of pigs had a tail lesion.

With the baseline parameters, and a 5.6% prevalence scenario, the financial loss was approximately €1.80 per finished pig. When the prevalence was at 10%, the financial loss was approximately €3.20 per finished pig (Figure 7).

## 4 Discussion

The results suggest that the desire to achieve good financial results by producing healthy pig with high welfare motivated Finnish farmers in addressing tail biting. Besides animal welfare and financial factors, a sense of success in producing high-quality pigs was one of the most motivating factors for managing tail biting. The interviewed farmers were very or fairly motivated to address tail biting when they observed it on their own farms.

Unlike in many other countries, tail docking has never been widely applied in Finland. Most of the farmers producing weaner pigs considered that tail docking should not be allowed, even though every second of them found management measures difficult to implement. Accordingly, Menegon et al. (1) have pointed out that tail docking is not a solution but merely an excuse when one should react to the other problems arising in production.

Interviewees from both farms producing weaner and finishing pigs considered tail biting to be a rather severe animal welfare issue. The risk of tail biting was considered to increase especially because of undesired changes occurring in the housing conditions or feeding of pigs, when there was a lack of enrichment and if there was low space allowance, particularly toward the end of the weaner rearing phase. These results are in line with earlier findings of D’Eath et al. (3) of risk factors causing tail biting. Respondents estimated that tail biting increases the risk of diseases, medication needs, and the removal of pigs, as well as requiring additional work on the farm. Longer rearing time of bitten pigs was estimated to increase production costs and lead to issues with space allowance, which, in turn, increase the risk of tail biting.

According to the interviewees, financial losses incurred by tail biting are caused not only by an increase in production costs but also because of lower-quality pigs and the resulting tail reduction in the price of weaned pigs, as well as carcass rejections, which may reduce both the quantity of meat sold and the price of pigmeat. In particular, farmers producing weaner pigs considered tail biting to be an economic issue.

Even though the available research data, training, or support for the systematic management of tail biting did not motivate the interviewees quite as much as factors related to the health and welfare of pigs and economic factors, they could help improve farm-level knowledge of how to manage tail biting. Training and support for both farmers and farm workers could be especially useful on farms producing weaner pigs, as these farmers considered tail biting to be a more severe welfare issue and found managing tail biting more difficult than farms producing finishing pigs.

Improving the living environment of the pig and the welfare of the pigs depends on the financial resources of the farm. Although additional space or workforce would be useful in managing tail biting, it may not be possible because of the poor profitability of production. Because tail biting both causes additional costs and reduces profits, it also affects the profitability of production. Indeed, various financial incentives are perceived as important in reducing tail biting.

The financial incentives that most motivated Finnish farmers producing weaner pigs to address tail biting were the higher prices obtained for intact-tailed pigs, an increase in the price of pigmeat, and financial support for implementing management measures. Finishing farms were most motivated by the reduction in carcass condemnations and the increase in price of pigmeat.

The discussion about incentives relates to what kind of subsidies could most effectively guide farmers’ choices. Input subsidies could be considered more effective in achieving the specific goals of sustainable agriculture, such as reducing chemical overuse or practices that compromise animal welfare, as they can directly target wanted or unwanted practices. Output subsidies, by contrast, are more effective for increasing total production and farm income [see, e.g., (14–17)]. However, tail biting is a multifactorial problem, and measures to control the risk of tail biting vary from case to case. Many interviewees raised the view that rather than supporting measures to manage tail biting, the outcome, i.e., the production of intact-tailed pigs, should be rewarded. This finding would also be in line with studies suggesting that it is economical more effective to reward for expected outcomes than to subsidize input use because rewarding for the end result enables the farmer to choose those management measures that are best-suited to his/her farm. In Finland, there is a price deduction for weaner pigs with tail lesions, whereas in some countries there is a reward for intact-tailed pigs. These aspects can be used to develop further incentives to reduce the risk of tail biting.

Our study shows that tail biting in weaner pig production can cause a loss of up to approximately €32 per bitten pig, taking into account possible consequential effects observed in the finishing phase, reduced growth, treatment of the tail-damaged pig, and various income losses and additional costs. The economic significance of tail biting is affected by the severity of tail biting; more severe and prevalent lesions are likely to lead to higher treatment and labor costs, and a higher risk of financial losses due to mortality and impaired growth rate per produced pig. Taylor et al. (18) suggested that tail biting can be expressed in three different descriptive types, namely, ‘two-stage’, ‘sudden-forceful’ and ‘obsessive’, each of which may have different motivational bases among pigs. Obsessive tail biting was characterized to result in mild to severe wounds, including partial or full amputation of the tail. Sudden-forceful tail biting was characterized by mild to severe bite marks on the tail, bleeding wounds, and two-stage tail biting with few if any lesions observed. As tail biting is manifested differently in these cases, the resulting financial losses can also be different. In addition, tail biting can be of the epidemic type (19). In this case, the outbreak can escalate quickly in the pen. In our study, a simulation was conducted to elaborate on the financial losses caused by tail biting; however, different types of tail biting were not explicitly simulated. However, our scenarios resemble sudden-forceful or obsessive tail types of tail biting as well as epidemic tail biting. In addition, we presented a scenario of escalated tail biting in which a very high percentage of pigs were assumed to have tail lesions, resulting in elevated financial losses for the farmer. This could be the case of very obsessive, epidemic tail biting.

The most important financial costs of tail biting were associated with an increased risk of pig mortality, medication and care for the weaned pigs, impaired growth and deductions in the pig price due to tail lesions when the pig is sold to the finisher farm. Pig mortality results both in the loss of pig’s value and the cost of disposing of dead pigs. Taking into account the incidence of tail lesions in the weaner rearing phase, the cost is approximately €1.80 per pig produced. Our estimated impacts fall within the range of losses reviewed by Niemi and Stevenson (2). However, the economic significance of tail biting on the weaner production farm depends heavily on how commonly tail biting occurs and how severe the lesions are.

Addressing tail biting is important not only for promoting animal welfare and economic viability of pig farming but also for the image of the pig industry. Interviewees found the pig industry’s good image among consumers to motivate them to manage tail biting as it was considered to enhance animal welfare more broadly. On the European level, public concern about pig welfare has largely focused on tail docking (3). Similarly, pig production without tail docking was considered an image benefit by Finnish interviewees. As consumer awareness of animal welfare issues is increasing (20), the good image of industry and operators is important for the development of pig production in the future.

This study focused on the financial effect of tail biting and on factors motivating producers to manage tail biting on Finnish pig farms. As the consequences of tail biting can be substantial, it is important that farmers are informed about the potential financial losses and impairment of animal welfare in the event of tail biting occurrences as well as possible means to mitigate and prevent tail biting. Even though the results on motivational factors are based on Finnish pig farming, they may be useful when developing incentives for tail biting prevention also in other countries.

## Data Availability

The raw data supporting the conclusions of this article will be made available by the authors, without undue reservation.
