# Supplementary material for: Motivational factors and financial incentives to mitigate tail biting in pig production in Finland
Source: Front Vet Sci. 2026 Aug 19;13:1860638. doi: 10.3389/fvets.2026.1860638 (PMC13533822; doi:10.3389/fvets.2026.1860638)
Supplement: Supplementary file 2 [file Supplementary_file_2.docx]

Appendix 2.

**Figure 3.** Weaner pig farmers’ (N=10) views on how much different factors related to influence of third parties and obligations would motivate them to intervene tail biting (1=does not motivate, 7=motivates very much).

**Figure 4.** Finishing pig farmers’ (N=15) views on how much different factors related to influence of third parties and obligations would motivate them to intervene tail biting (1=does not motivate, 7=motivates very much).
