# Supplementary material for: Motivational factors and financial incentives to mitigate tail biting in pig production in Finland
Source: Front Vet Sci. 2026 Aug 19;13:1860638. doi: 10.3389/fvets.2026.1860638 (PMC13533822; doi:10.3389/fvets.2026.1860638)
Supplement: Supplementary file 3 [file Table_1.docx]

Supplementary Material

Supplementary Table 1. Information and parameters used in the computational model.

| **Parameter** | **Value** | **Source** |
| --- | --- | --- |
| Biological parameters |  |  |
| Average number of litters produced per sow | 3,8 | Assumption based on production data |
| Number of liveborn piglets, piglets/sow | 17,0 | Assumption based on production data |
| Piglet mortality during lactation, % | 13,0 % | Assumption based on production data |
| Piglet mortality at weaner rearing, %, intact tails | 3,5 % | Assumption based on production data |
| Piglet mortality at weaner rearing, %, damaged tails | 10,1 % | Assumption based on production data |
| Mortality during fattening, % | 1 % | Assumption based on production data |
| Average weight at weaning, kg | 6,90 | Assumption based on production data |
| Piglet's age at weaning, days | 28,00 | Assumption based on production data |
| Average weight of piglets when leaving weaner rearing phase, kg | 28,00 | Assumption based on production data |
| Piglet's age when leaving weaner rearing phase, days, intact tails | 76,84 | Farm data |
| Piglet's age when leaving weaner rearing phase, days, damaged tails | 72,00 | Farm data |
| Feed conversion ratio at weaner rearing, FU/kg gain | 1,54 | Assumption based on production data |
| Feed conversion ratio during finishing, kg feed/kg gain | 2,3 | Assumption based on production data |
| Average slaugter weight, kg live weight | 120,3 | Assumption based on production data |
| Average daily gain during fattening, kg/d | 1031,5 | Assumption based on production data |
| Red meat percentage, % | 59,5 | Assumption based on production data |
| Carcass condemnations, % carcasses rejected, intact tails | 3,9 % | Calculated based on Valros et al. (2004, 2020) |
| Carcass condemnations, % carcasses rejected, damaged tails | 3,0 % | Calculated based on Valros et al. (2004, 2020) |
| Price parameters |  |  |
| Price of pigmeat, €/kg | 2,12 | www.luke.fi/stat |
| Price reduction for piglets with tail damage, €/piglet | 5 | Information obtained from meat companies |
| Welfare compensation for enhanced farrowing conditions, €/sow/year | 64 | Finnish Food Authority |
| Animal welfare compensation for enhanced housing, €/fattening pig | 17,70 | Finnish Food Authority |
| Feed price, lactating sows, €/FU | 0,37 | Information collected from feed suppliers |
| Feed price, idle or lactating sows, €/FU | 0,30 | Information collected from feed suppliers |
| Feed price, suckling piglets, €/kg | 0,49 | Information collected from feed suppliers |
| Feed price, piglets at weaner rearing, €/FU | 0,51 | Information collected from feed suppliers |
| Feed price, fattening pigs, €/FU | 0,23 | Information collected from feed suppliers |
| Price of gilt, €/gilt | 350,00 | Information obtained from meat companies |
| Cost of artificial insemination, €/insemination | 5,65 | Information obtained from semen supplier |
| Cost of treating tail bitten pigs, €/bitten pig. | 12,03 | Data updated from Niemi et al. (2021) |
| Cost of labor input, €/h | 16,70 | Calculated based on a labor market agreement for agricultural workers and social security fees. |
| Carcass rendering costs, €/dead piglet | 4,27 | Calculated by using information from Honkajoki rendering company |
| Carcass rendering costs, €/dead fattening pig | 16,00 | Calculated by using information from Honkajoki rendering company |
| Price of electricity, €/MWh | 0,70 | Local electicity supplier |
| Price of water, €/m^3^ | 1,00 | Local water department |
| Maintenance and insurance, % of investment cost | 1,5 % | Assumption, farm advisors |
| Overhead, % of investment cost | 4,0 % | Assumption, farm advisors |
| Michellaneous costs at pig fattening, €/sow | 2,90 | Author's own calculation |
| Michellaneous costs at piglet production, €/sow | 80,00 | Author's own calculation |
| Annual interest rate, % | 5 % | Assumption, farm advisors |
| Investment cost, €/sow space unit | 3600,00 | https://www.finlex.fi/fi/lainsaadanto/2023/608 |
| Investment cost, €/fattening pig space unit | 840,00 | https://www.finlex.fi/fi/lainsaadanto/2023/608 |

**References**

Niemi JK, Edwards SA, Papanastasiou DK, Piette D, Stygar AH, Wallenbeck A and Valros A. Cost-Effectiveness Analysis of Seven Measures to Reduce Tail Biting Lesions in Fattening Pigs. Front. Vet. Sci. (2021) 8: 682330. doi: 10.3389/fvets.2021.682330

Valros A, Ahlstrom S, Rintala H, Hakkinen T, Saloniemi H. The prevalence of tail damage in slaughter pigs in Finland and associations to carcass condemnations. Acta Agric Scand Section A-Anim Sci. (2004) 54:213–9. 10.1080/09064700510009234

Valros A, Välimäki E, Nordgren H, Vugts J, Fàbrega E, Heinonen M. Intact tails as a welfare indicator in finishing pigs? Scoring of tail lesions and defining intact tails in undocked pigs at the abattoir. Front Vet Sci. (2020) 7:405. 10.3389/fvets.2020.00405
